# RIS Wireless Network Optimization Based on TD3 Algorithm in Coal-Mine Tunnels

**DOI:** 10.3390/s25196058

**Published:** 2025-10-02

**Authors:** Shuqi Wang, Fengjiao Wang

**Affiliations:** School of Communication and Information Engineering, Xi’an University of Science and Technology, Xi’an 710600, China; 15205848573@163.com

**Keywords:** coal-mine tunnel communication, reconfigurable intelligent surface, twin delayed deep deterministic policy gradient, sum rate

## Abstract

As an emerging technology, Reconfigurable Intelligent Surfaces (RIS) offers an efficient communication performance optimization solution for the complex and spatially constrained environment of coal mines by effectively controlling signal-propagation paths. This study investigates the channel attenuation characteristics of a semi-circular arch coal-mine tunnel with a dual RIS reflection link. By jointly optimizing the base-station beamforming matrix and the RIS phase-shift matrix, an improved Twin Delayed Deep Deterministic Policy Gradient (TD3)-based algorithm with a Noise Fading (TD3-NF) propagation optimization scheme is proposed, effectively improving the sum rate of the coal-mine wireless communication system. Simulation results show that when the transmit power is 38 dBm, the average link rate of the system reaches 11.1 bps/Hz, representing a 29.07% improvement compared to Deep Deterministic Policy Gradient (DDPG). The average sum rate of the 8 × 8 structure RIS is 3.3 bps/Hz higher than that of the 4 × 4 structure. The research findings provide new solutions for optimizing mine communication quality and applying artificial intelligence technology in complex environments.

## 1. Introduction

With the widespread application of 5G technology in underground coal-mine communications, the construction of “smart mines” with the goals of visualization, unmanned operation, informatization, and intelligence is gradually being realized [1,2,3]. To effectively address the challenges of signal quality and coverage blind spots in the complex underground propagation environment, novel superconducting reflective surface technology with passive controllable signal characteristics has been introduced into mine safety production and information exchange processes. By dynamically adjusting signal-propagation characteristics, this technology constructs an adaptive underground signal coverage network, enabling the controllability of random and uncontrollable complex channels [4,5,6,7]. Currently, research on reflector-assisted wireless communication technology in mines can be primarily categorized into two aspects: the first focuses on the hardware structure design of reflectors [8,9,10] and the study of mine channel enhancement mechanisms using traditional algorithms [11,12,13]; the second focuses on the autonomous optimization design of mine reflectors based on intelligent algorithms such as deep learning.

Reference [14] investigates the field-strength superposition principle of multiple Reconfigurable Intelligent Surfaces (RIS) in tunnel scenarios. By constructing a vector model, it studies the signal field-strength characteristics of the RIS receiving end at a frequency of 28 GHz in subway tunnels and provides a deployment scheme for multiple RIS within a limited dynamic distribution space. Reference [15] focused on wireless communication in mine tunnels, specifically investigating non-line-of-sight propagation at the 28 GHz frequency band. It employed 3D ray-tracing simulations to model mine tunnel environments and evaluated the effectiveness of different RIS deployment modes—reflective and absorptive—in enhancing communication capacity under high path loss and multipath interference conditions. In [16], a joint deployment strategy for 5G-R base stations assisted by RIS is proposed, providing a wireless coverage solution for mountainous railways using RIS. It studies system gains using an optimized SINR criterion and the Charnes–Cooper solution method, and investigates the signal compensation characteristics of a 100-unit array RIS at the 930 MHz frequency band. In [17], a joint beamforming scheme for RIS-assisted multi-user MIMO systems based on deep learning is proposed, investigating system performance and data rates under various RIS array configurations. However, this study does not fully consider multi-RIS deployment scenarios or multi-hop signal propagation. In [18,19], multi-hop RIS-assisted joint communication and sensing (JCAS) methods are employed to enhance the energy efficiency and overall sensing rate of JCAS access points in underground coal mines. In [20], an error-rate detection method for mine RIS systems based on the Parallel Interference Cancellation (PIC) algorithm is proposed and its error characteristics are analyzed. However, this study only targets ideal rectangular tunnels and does not consider the complex tunnel structures in real-world scenarios. In [21], a RIS-NOMA mine communication system model based on wireless local area network noise interference is proposed, evaluating system transmission reliability by analyzing the interruption probability. In [22], an underground communication system model based on the Nakagami-g fading channel model and RIS signal-propagation model is constructed, with preliminary channel estimation performed using the least squares (LS) algorithm, and the channel estimation results optimized using an octave convolution (OCT) neural network. In [23], the reconfigurable characteristics of passive RIS beams are implemented through the principles of passive coding and splicing, suitable for coal-mine tunnels with different turning angles.However, traditional algorithms exhibit slower computation speeds and lower efficiency when handling complex environments. Therefore, adopting more efficient intelligent algorithms is particularly crucial for addressing the intricate scenarios encountered in coal-mine communication systems.

Reference [24] introduces RIS into the Vehicle-to-Everything (V2X) communication system for mines. By integrating meta-heuristic optimization algorithms with deep learning techniques and incorporating an error-correction strategy, it optimizes the RIS phase configuration to enhance channel gain.Reference [25] utilizes DRL to obtain the optimal RIS-assisted computational unloading strategy in dynamic mining environments, proposing a RIS-assisted computational unloading scheme based on Deep Deterministic Policy Gradient (DDPG) to jointly optimize the phase shift and unloading rate of RIS components, thereby maximizing the utility of mining IoT devices, improving energy efficiency, and reducing computational latency. In [26], RIS is used to optimize signal quality and maximize spectral efficiency in high-speed railway tunnels, proposing an algorithm combining Long Short-Term Memory (LSTM) and DDPG, namely LSTM-DDPG, to address this issue. In [27,28,29,30], the channel model and fading for RIS-assisted rectangular mines are derived, the feasibility of RIS in mine communications is verified, and the DDPG algorithm is used to optimize the system and rate in rectangular tunnels.DDPG and LSTM-DDPG have achieved some progress in RIS optimization, but they still face challenges in handling noise issues in complex environments and multi-hop RIS deployments. The DDPG algorithm suffers from overestimation problems, which may lead to inefficient and unstable policy optimization. Therefore, there is an urgent need to propose more efficient intelligent algorithms.

The existing literature primarily focuses on simple rectangular tunnel scenarios assisted by RIS, without addressing more complex mine tunnel geometric structures. The main contributions of this paper are summarized in the following three key aspects:

(1) This paper proposes for the first time a novel RIS channel propagation model specifically designed to model complex semi-circular vaulted mine structures. This model accounts for the geometric characteristics of mine tunnels, filling a gap in the existing literature that has only addressed simple rectangular tunnels.
(2) To overcome the noise issues in the existing Twin Delayed Deep Deterministic Policy Gradient (TD3) algorithm, this paper proposes an improved TD3 algorithm—TD3 with Noise Fading. By dynamically adjusting the standard deviation of Ornstein–Uhlenbeck (OU) noise, this paper effectively mitigates the negative impact of noise on algorithm performance, enhancing the stability and efficiency of the optimization process.
(3) The proposed TD3-NF algorithm jointly optimizes the base-station beamforming matrix and the RIS phase-shift matrix to maximize the system link rate. The paper also analyzes the impact of base-station transmit power and the number of RIS units on the link rate, further explores the influence of key neural network parameters on algorithm performance, and provides new insights for the design of coal-mine RIS communication systems.

The remainder of this paper is structured as follows: Section 2 introduces the system model, Section 3 presents the research questions and proposes corresponding solutions, Section 4 evaluates the proposed approach through simulation, and Section 5 concludes the paper.

## 2. System Model

**Figure 1:**
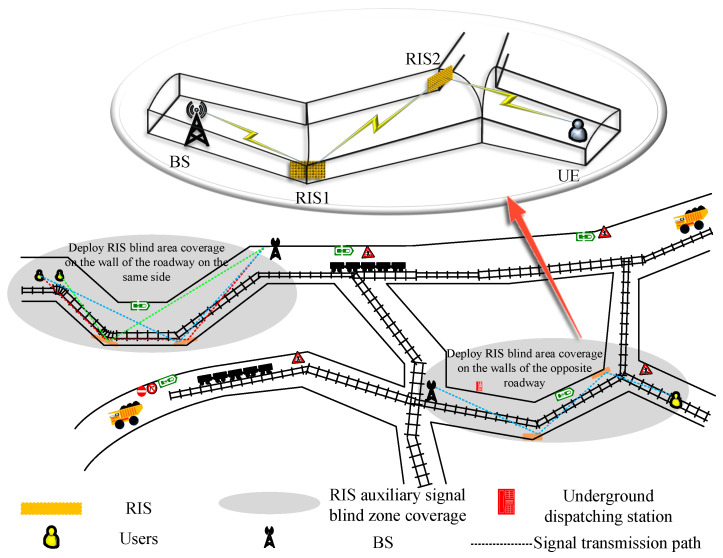
Dual RIS-assisted coal-mine communication system.

Figure 1 shows the simulated distribution of multi-branch mine tunnels. The tunnel geometric cross-section consists of a combination of rectangular tunnel structures and slightly curved semi-circular arched roofs. The figure illustrates two RIS-assisted tunnel scenarios: one is a communication system model assisted by RIS on the same side wall, and the other is a communication system model assisted by RIS on the opposite side wall. To enhance the feasibility of the system model design, the study fully considers the curved and narrow conditions of the mine tunnels. It is important to note that RIS is used for signal coverage in underground environments, so the research focuses solely on the signal-propagation characteristics of RIS-assisted mine tunnels under Non-Line-of-Sight (NLoS) conditions. Due to the complex long-distance propagation in confined spaces, the study does not consider the impact of Line-of-Sight (LoS) propagation on the system.

Consider a dual RIS-assisted multi-user coal-mine communication system with obstacles. The system is specified as consisting of a base station (BS) with *M* antennas and two RISs composed of $N_{r}\left(r\in \left\{1,2\right\}\right)$ reflective units.Here, we assume that the two RIS systems have an equal number of reflectors (i.e., $N_{1}=N_{2}$). This ensures dimensional matching in matrix operations, thereby guaranteeing the validity of the formula and the mathematical consistency of the system model. Furthermore, setting the reflector count equal simplifies the signal-processing model and ensures symmetry during signal optimization. RISs deployed on the curved walls of the mine tunnels, RIS1 near the BS end, RIS2 near the UE end and *K* single-antenna users (UE). The channels from BS to RIS1 and RIS2 are denoted as $H_{n_{1}m}\in C^{N_{1}\times M}$ and $H_{n_{2}m}\in C^{N_{2}\times M}$, respectively, the channels from RIS1 and RIS2 to UE are denoted as $H_{n_{1}k}\in C^{N_{1}\times K}$ and $H_{n_{2}k}\in C^{N_{2}\times K}$ respectively, and the channel from RIS1 to RIS2 is denoted as $F\in C^{N_{2}\times N_{1}}$.

### 2.1. Coal-Mine Tunnel Modeling

Define the reflection coefficient matrix of RIS as shown in Equation (1):

(1) $$\Phi_{r}=diag\left(\beta \left(\theta_{N_{r}}\right)e^{j\theta_{N_{r}}}\right),\theta_{n_{r}}\in \left[0,2\pi \right)$$

where $\theta_{n_{r}}\in \left[0,2\pi \right)$ is the phase change caused by the r−th reflection element of the n−th RIS, and $\beta (\theta_{n_{r}})$ is an amplitude function based on the $\theta_{n_{r}}$ phase change:

(2) $$\beta \left(\theta_{n_{r}}\right)=\left(1-\beta_{\min}\right){\left(\frac{\sin\left(\theta_{n_{r}}-\mu \right)+1}{2}\right)}^{\alpha }+\beta_{\min}$$

where μ represents the horizontal offset of the phase shift; and α controls the steepness of the function curve. $\beta_{\min},\mu \geq 0,\alpha \geq 0$ depends on the hardware implementation constants of the RIS. If the signal undergoes ideal reflection on the RIS, then ${\left|\phi_{n}\right|}^{2}=1$, i.e., $\beta_{\min}=1$, or α=0.

The aggregated channel from the BS to the user *k* is shown in Equation (3):

(3) $$H_{k}=\underset{\mathrm{dual}\;\text{-}\;\mathrm{reflection}\;\mathrm{link}}{\underbrace{H_{n_{2}k}\Phi_{2}F\Phi_{1}H_{n_{1}m}}}+\underset{\sin\mathrm{gle}\;\text{-}\;\mathrm{reflection}\;\mathrm{link}}{\underbrace{H_{n_{1}k}\Phi_{1}H_{n_{1}m}+H_{n_{2}k}\Phi_{2}H_{n_{2}m}}}$$

Due to the wide-frequency spectrum characteristics of electrical noise, electrical noise that affects radio signals is regarded as a pulse interference response. Therefore, underground noise can be represented by an independent and identically distributed Bernoulli–Gaussian process:

(4) $$W=w_{\mathrm{bg}}+w_{\mathrm{imp}}=w_{\mathrm{bg}}+B\cdot G_{a}$$

where $w_{\mathrm{bg}}∼N\left(0,\sigma^{2}\right)$ is additive Gaussian white noise, pulse interference noise $w_{\mathrm{imp}}=B\cdot G_{a}$ is Gaussian noise with random pulses, B is a Bernoulli random process with mean 0 and variance 1, taking values 0 or 1, $G_{a}∼N\left(0,\sigma_{\mathrm{imp}}^{2}\right)$.

The signal received from the k−th user can be expressed as shown in Equation (5):

(5) $$y_{k}=P_{k}H_{k}g_{k}s_{k}+P_{n}H_{k}\sum_{j=1,j\neq k}^{K}g_{j}s_{j}+w_{k}$$

where the first term represents the expected signal of the k−th user, the second term represents the interference caused by the signals of all other users (n≠k) to the k−th user, i.e., co-channel interference (CCI), $s_{k}∼CN\left(0,\sigma^{2}\right)$ is the data symbol transmitted by the base station to the user *k*, $g_{k}\in C^{N\times 1}$ is the transmission beamforming vector of the base station to user *k*, and *P* is the transmission power of each signal. Therefore, the signal-to-interference-plus-noise ratio (SINR) of the k−th user in the system can be expressed as shown in Equation (6):

(6) $$\beta_{k}=\frac{P_{k}|H_{k}g_{k}{|}^{2}}{P_{n}\sum_{n.n\neq k}^{K}|H_{k}g_{j}{|}^{2}+\sigma^{2}+p\cdot \sigma_{\mathrm{imp}}^{2}}$$

where *p* is the probability that B equals 1. Then, the total link rate in the system (in units of bps/Hz) can be expressed as shown in Equation (7):

(7) $$C=\sum_{k=1}^{K}\log_{2}\left(1+\beta_{k}\right)$$

### 2.2. Signal-Propagation Path Analysis

**Figure 2:**
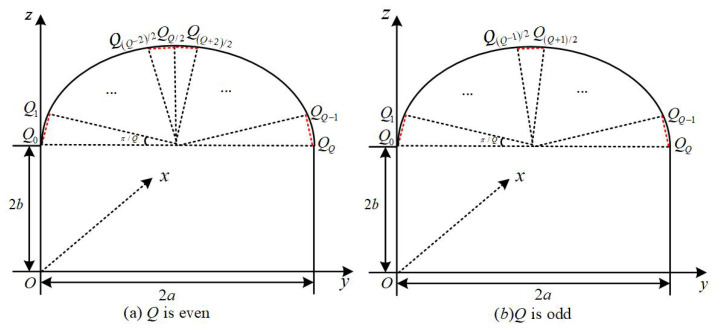
Semi-circular arch coal-mine tunnel roof decomposition diagram.

The study uses the mirror method to predict the propagation characteristics of signals. First, it is necessary to obtain the effective reflection surface required for the mirror method. As shown in Figure 2, the arch is divided into *Q* equal parts. Regardless of whether *Q* is odd or even, the Y-axis coordinate and the Z-axis coordinate of any coordinate point $Q_{q}=\left(x_{q},y_{q},z_{q}\right)$ can be expressed as shown in Equation (8):

(8) $$y_{q}=a-a\cos\left(\frac{q\pi }{Q}\right),z_{q}=b+a\sin\left(\frac{q\pi }{Q}\right)$$

The q−th small plane of the arch is determined by two adjacent points $\left(y_{q},z_{q}\right)$ and $\left(y_{q-1},z_{q-1}\right)$. The plane equation of the q−th small plane is shown in Equation (9):

(9) $$\frac{\left(y-y_{q}\right)\left(z_{q}-z_{q-1}\right)}{y_{q}-y_{q-1}}-z+z_{q}=0$$

The plane equation of the coal-mine wall surface is shown in Equation (10):

(10) $$\left\{\begin{array}{cc} \frac{\left(y-y_{q}\right)\left(z_{q}-z_{q-1}\right)}{y_{q}-y_{q-1}}-z+z_{q}=0 & \mathrm{Top}\;\mathrm{plate}\\ x=\pm a & \mathrm{Left}/\mathrm{right}\;\mathrm{side}\;\mathrm{walls}\\ z=0 & \mathrm{Bottom}\;\mathrm{plate} \end{array}\right.$$

Specify the coordinates of the launch point $A(x_{0},y_{0},z_{0})$, the coordinates of the receiving point $B(x_{1},y_{1},z_{1})$, the coordinates of the reflection point C(x,y,z), and the equation of the reflection plane *m* is $a_{1}x+a_{2}y+a_{3}z+a_{4}=0$, constructed in the Cartesian coordinate system shown in Figure 2. The mirror point $M(x_{m},y_{m},z_{m})$ can be expressed as shown in Equation (11):

(11) $$\left\{\begin{array}{c} x_{m}=x_{0}\\ y_{m}=\left[\left(a_{3}^{2}-a_{2}^{2}\right)y_{0}-2a_{2}a_{3}z_{0}-2a_{2}a_{4}\right]/\left(a_{2}^{2}+a_{3}^{2}\right)\\ z_{m}=\left[\left(a_{2}^{2}-a_{3}^{2}\right)z_{0}-2a_{2}a_{3}y_{0}-2a_{3}a_{4}\right]/\left(a_{2}^{2}+a_{3}^{2}\right) \end{array}\right.$$

According to the line connecting the mirror point and the receiving point MB and the reflection plane intersecting at the reflection point, we have:

(12) $$\frac{x_{m}-x}{x_{1}-x}=\frac{y_{m}-y}{y_{1}-y}=\frac{z_{m}-z}{z_{1}-z}=u$$

From this, we can obtain:

(13) $$x=\frac{x_{m}-ux_{1}}{1-u},y=\frac{y_{m}-uy_{1}}{1-u},z=\frac{z_{m}-uz_{1}}{1-u}.$$

The reflection point is also located on the reflection plane, so it satisfies the reflection plane equation, giving us:

(14) $$u=\frac{a_{1}x_{m}+a_{2}y_{m}+a_{3}z_{m}+a_{4}}{a_{1}x_{1}+a_{2}y_{1}+a_{3}z_{1}+a_{4}}$$

The coordinates of the reflection point can be obtained from Equations (13) and (14).

In a semi-circular arch-shaped mine tunnel, the signal undergoes more than three reflections, resulting in significant energy attenuation. Therefore, considering only the first three reflections is sufficient to fully reveal the signal-propagation behavior. Thus, the upper limit for the number of reflections is set to three [31,32]. The number of reflection lines depends on the number of reflective surfaces ξ in the tunnel, where reflective surfaces include the tunnel walls and RIS. A single reflection line can have up to ξ lines, with each reflection line involving one reflective surface; a secondary reflection line can have up to ξ(ξ−1) lines, with any two different reflective surfaces forming a secondary reflection line; a tertiary reflection line can have up to $\xi {\left(\xi -1\right)}^{2}$ lines, with each tertiary reflection line passing through three different reflective points, and adjacent reflective points cannot be on the same reflective surface, but the first and third reflective points can be on the same reflective surface. In this process, the RIS, as a new type of controllable reflective surface, collaborates with traditional reflective surfaces to construct a multipath propagation environment. Therefore, the total effective scattering paths are shown in Equation (15):

(15) $$\xi +\xi \left(\xi -1\right)+\xi {\left(\xi -1\right)}^{2}$$

Assuming that the signal passes through *n* reflection points $C_{1},C_{2},\ldots ,C_{n}$ from the transmission point to the reception point, the path length of the signal propagation can be expressed as: $L=\sum_{i=1}^{n}p_{i},i=1,2,\ldots ,n$; where $p_{i}$ represents the distance from the i−th reflection point $C_{i}$ to the (i+1)−th reflection point $C_{i+1}$, and the reflection points include the position of RIS. In the case of dual RIS, the length of the reflection path changes with the number of RIS, so it is necessary to calculate the distance between each reflection point step by step.

According to Fresnel’s law, the reflection coefficients of vertically polarized waves and horizontally polarized waves can be expressed as shown in Equation (16) and (17):

(16) $$R_{⊥}=\frac{\cos\theta_{i}-\sqrt{\varepsilon_{r}-\sin^{2}\theta_{i}}}{\cos\theta_{i}+\sqrt{\varepsilon_{r}-\sin^{2}\theta_{i}}}$$

(17) $$R_{\|}=\frac{\varepsilon_{r}\cos\theta_{i}-\sqrt{\varepsilon_{r}-\sin^{2}\theta_{i}}}{\varepsilon_{r}\cos\theta_{i}+\sqrt{\varepsilon_{r}-\sin^{2}\theta_{i}}}$$

where $\varepsilon_{r}$ is the relative dielectric constant of the tunnel wall. Assuming that the roughness distribution of the tunnel wall follows a Gaussian distribution with a mean of 0 and a variance of *h* and $\rho_{r}$ is the roughness loss factor, the roughness loss coefficient caused by the rough surface of the tunnel wall is:

(18) $$\rho_{⊥}=\rho_{r}R_{⊥},\rho \|=\rho_{r}R_{\|}$$

where $\rho_{r}=\exp\left[-8{\left(\frac{\pi h\cos\theta_{i}}{\lambda }\right)}^{2}\right]$. Multiple reflections of the signal on both sides of the tunnel and the top and bottom plates will cumulatively affect the total reflection coefficient. Assuming the ray undergoes *m* reflections at the side walls and *n* reflections at the top and bottom plates (i.e., experiencing *m* reflections of horizontally polarized waves followed by *n* reflections of vertically polarized waves at the top and bottom plates), the reflection coefficient at this point can be expressed as shown in Equation (19):

(19) $$\omega_{p}=\left\{\begin{array}{c} R_{⊥}^{m}R_{\|}^{n}\rho_{r}^{m+n}\\ R_{\|}^{m}R_{⊥}^{n}\rho_{r}^{m+n} \end{array}\right.$$

## 3. Joint Design of Base-Station Beamforming and RIS Phase Shifting Based on TD3-NF

### 3.1. Optimization of Beamforming Matrix and RIS Phase Shift Matrix

The power of the multi-antenna BS transmission signal is subject to the maximum transmission power constraint, expressed as shown in Equation (20):

(20) $$E\left\{Tr\left(GG^{H}\right)\right\}\leq P_{\max}$$

where $P_{\max}$ is the maximum transmission power at BS, E{·} represents the statistically expected value, and Tr(·) represents the trace of the matrix.

In order to maximize the total link rate of the system by optimizing G and Φ, define the optimization problem:

(21) $$\begin{array}{cc} \; & \max\sum_{k=1}^{K}\log_{2}\left(1+\beta_{k}\right)\\ \; & s.t.\;\mathrm{Tr}\left(GG^{H}\right)\leq P_{\max}\\ \; & \;\;|\phi_{n}{|}^{2}=1\\ \; & \;\;0\leq \theta_{n}<2\pi ,\;n=\left\{1,2,\ldots ,N\right\} \end{array}$$

Due to the non-convexity of the objective function and the complexity of the constraints, traditional mathematical optimization methods require a large number of iterative calculations, resulting in high computational complexity. Therefore, this paper adopts a framework based on the TD3 algorithm to solve the problem in Equation (21) and obtain feasible G and Φ under the premise of satisfying all feasible constraints.

### 3.2. TD3-NF Algorithm Design

The TD3 algorithm is an improvement on the DDPG algorithm, both of which are used for continuous control problems. Compared to DDPG, TD3 introduces two key innovations: dual updates of the target network and an action delay update mechanism to reduce estimation errors during training.

**Figure 3:**
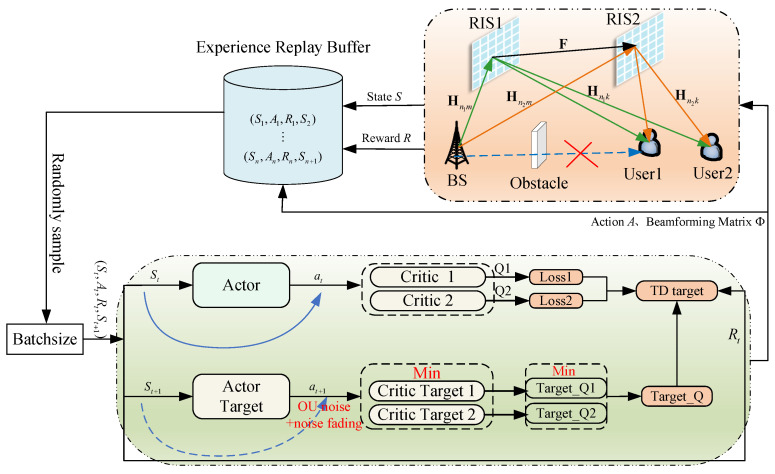
TD3-NF algorithm block diagram.

In standard TD3, Ornstein–Uhlenbeck (OU) noise is used to increase the randomness of action selection to promote exploration. However, the standard deviation of OU noise is typically fixed, which may result in an overly prolonged exploration phase or excessive noise persisting in the later stages of training, thereby impairing the model’s exploitation efficiency. To address this issue, this paper proposes the TD3-NF algorithm to solve problem in Equation (21). This algorithm balances exploration and exploitation by gradually reducing the standard deviation of the noise, i.e.,

(22) $$\sigma_{t}=\max\left(\sigma_{t-1}\cdot \gamma_{s},\sigma_{\min}\right)$$

where $\gamma_{s}$ is the noise decay rate, $\sigma_{\min}$ is the minimum standard deviation of noise, and $\sigma_{t-1}$ is the standard deviation of noise in the previous step. This decay strategy allows the algorithm to conduct extensive exploration in the early stages and gradually transition to a more strategy-dependent utilization phase as training progresses. The design process of problem in Equation (21) is shown in Figure 3.

Using CSI as input for the TD3-NF agent, generate the optimal G and Φ. The specific settings for state, action, and reward are as follows:

- State $s_{t}$: Transmit power at time *t*; channel matrices from BS to RIS1 and RIS2; channel matrices from RIS1 and RIS2 to UE; channel matrix from RIS1 to RIS2. The state space size is shown in Equation (23):
  (23) $$N_{i}=2K+2K^{2}+2MK+2\left(N_{1}+N_{2}\right)+2M\left(N_{1}+N_{2}\right)+2N_{1}N_{2}+2\left(N_{1}+N_{2}\right)K$$
- Action $a_{t}$: Composed of the beamforming matrix and phase shift matrix at time *t*; the size of the action space is shown in Equation (24):
  (24) $$N_{o}=2MK+2N_{1}+2N_{2}$$
- Reward $r_{t}$: The reward at time *t* is the value of the objective function in Equation (21):
  (25) $$r_{t}=\log_{2}\left(1+\beta_{k}\right)$$

The TD3-NF algorithm proposed in this paper follows the procedure below, as summarized in Algorithm 1.
**Algorithm 1.** TD3-NF algorithm**1** **Initial:** transmit beamforming matrix *G*, phase-shift matrix Φ, experience replay pool *E*, parameters of the Critic training network $w_{c_{1}},w_{c_{2}}$, parameters of the Actor training network $w_{a}$, parameters of the target network $w_{c_{1}}^{\prime },w_{c_{2}}^{\prime },w_{a}^{\prime }$**2** **Input:** channel matrices from BS to RIS1 and RIS2 $H_{h_{1}m},H_{h_{2}m}$; channel matrices from RIS1 and RIS2 to UE $H_{n_{1}k},H_{n_{2}k}$; channel matrix from RIS1 to RIS2 F**3** **Output:** Q-value function, optimal action a={G,Φ}**4** **for** *j=1 **to** J* **do****5**    Get the initial state $s_{0}$ **for** *t=1 **to** T* **do****6**       From the Actor training network $a_{t}$, $a_{t}=\pi \left(s_{t},w_{a}\right)$**7**       Based on the action $a_{t}$, get the state $s_{t+1}$ at the next moment, and get the immediate reward $R_{t+1}$ Store $\left(s_{t},a_{t},R_{t+1},s_{t+1}\right)$ into the experience replay pool *E***8**       A small batch of samples of size *W* is randomly drawn from the empirical replay pool *E***9**       Select two target Critic networks for Q-value prediction, $y_{1},y_{2}$**10**       Calculate TD Objectives $y_{\min}$**11**       Update Critic: $w_{c}\leftarrow w_{c}-\beta_{c}\cdot \nabla_{w_{c}}l\left(w_{c}\right)$**12**       Update Actor: $w_{a}\leftarrow w_{a}-\beta_{a}\cdot \nabla_{a}q\left(s_{t},a_{t};w_{c}\right)\nabla_{w_{a}}\pi \left(s_{t};w_{a}\right)$**13**       Update target networks: $w_{a}^{\prime }\leftarrow \tau w_{a}+\left(1-\tau \right)w_{a}^{\prime },\;0<\tau \ll 1$ $w_{c}^{\prime }\leftarrow \tau w_{c}+\left(1-\tau \right)w_{c}^{\prime },\;0<\tau \ll 1$**14**       Let $s_{t}=s_{t+1}$**15**    **end****16** **end**

**Figure 4:**
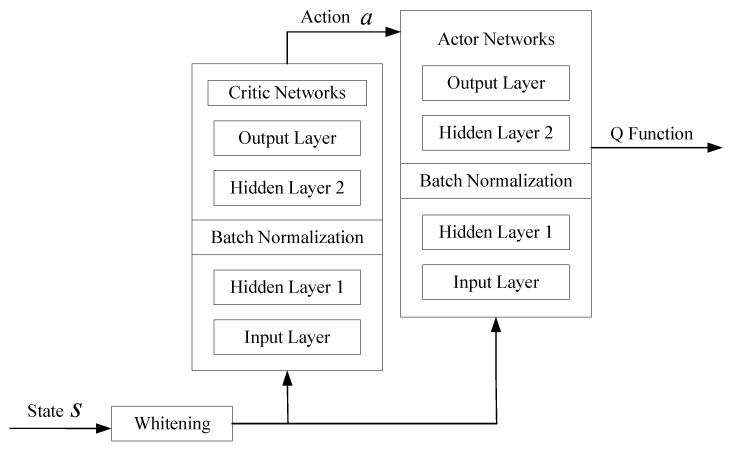
Neural network structure diagram.

As the number of training iterations increases, the agent continuously interacts with the environment to select the optimal actions, ultimately maximizing long-term returns. When training approaches convergence, the agent calculates the optimal beamforming matrix and RIS phase-shift matrix. The neural network structure designed is shown in Figure 4. The action network and evaluation network, as well as their respective target networks, are structurally similar, both consisting of four fully connected layers: an input layer, two hidden layers (with batch normalization applied in the hidden layers), and an output layer. Based on this, all layers of the deep neural networks in this paper use the tanh activation function, and all networks uniformly use the Adam optimizer. The learning rate in Adam is adaptively adjusted according to the gradient changes of each parameter, i.e., ${\beta_{a}}^{\left(t+1\right)}=\lambda_{a}{\beta_{a}}^{\left(t\right)}$, and ${\beta_{c}}^{\left(t+1\right)}=\lambda_{c}{\beta_{c}}^{\left(t\right)}$, where $\lambda_{a}$ and $\lambda_{c}$ are the decaying rates of the action network and critic network, respectively. $\beta_{c}$ is the learning rate for updating the Critic training network, and $\beta_{a}$ is the learning rate for updating the Actor training network.

During the training phase, the variables *L*, $Z_{0}$ and $Z_{l}$ represent the size of the training layer, the size of the input layer, and the number of neurons in layer *l*, respectively, and the number of learned samples is set to *B*. The computational complexity of each time step is $O(B\left(Z_{0}Z_{l}+\sum_{l=1}^{L-1}Z_{l}Z_{l+1}\right))$. During the training phase, each minibatch contains $N^{epi}$ events, each event is *T* time steps long, and each training model undergoes multiple iterations until convergence. Therefore, the total computational complexity of the training is expressed as: $O(N^{epi}B\left(Z_{0}Z_{l}+\sum_{l=1}^{L-1}Z_{l}Z_{l+1}\right))$. During the training phase, in addition to the forward propagation and backpropagation computations of the neural network, environmental simulation is required. This involves calculating the composite channel matrix for each user and performing matrix multiplication for RIS phase shifts. The computational complexity of channel calculation is $O\left(N_{r}\cdot K\right)$, where $N_{r}$ represents the number of reflectors in the RIS and *K* denotes the number of users. Additionally, the SINR for each user must be computed, introducing further computational overhead. During the simulation phase, the overall computational complexity increases to $O\left(N_{r}\cdot K\cdot J\right)$, where *J* corresponds to the number of SINR calculations performed per user.

## 4. Results

To validate the feasibility of the proposed TD3-NF-based algorithm for improving transmission rates in RIS-assisted semi-circular arch tunnels, this section conducts simulation experiments using numerical modeling, with Python as the programming language. First, an environment initialization function is constructed and initial values are set. An environment transition function is designed to describe the changes in the environment state after each action step. The neural network structure and parameters of the proposed TD3-NF-based algorithm are designed, and a DRL agent is constructed to interact with the environment. The coal-mine height is set to 6 m, width to 5 m, arch crown radius to 2.5 m, and the distances from the base station and receiver to the tunnel sidewall are 4.5 m and 2 m, respectively. The number of reflector array elements is 4×4, 4×8, 8×8, and the element size is $d_{x}=d_{y}$ = 5 cm. At 2.4 GHz, the dimensions of the RIS are 23.75×23.75 cm, 23.75×48.75 cm, and 48.75×48.75 cm, with a transmission distance of 200 m. The distance between the RIS and the base station is fixed at 50 m. In this study, we assume that the underground mine communication system employs a leaky feeder antenna configuration, a common antenna structure in complex environments such as mines that provides effective signal coverage. Signals are modulated using QPSK, with a transmission bit rate set at 1 Mbps, a beamwidth of 120°, and a standard time-division multiplexing (TDM) frame structure. The channel matrices $H_{n_{1}m}$, $H_{n_{2}m}$ and $H_{n_{1}k}$, $H_{n_{2}k}$ are implemented using ray tracing, where the total effective scattering paths are R=128, the gain of the receiving and transmitting antennas is 5 dBm, the roughness variance of the tunnel walls is h=0.01, and the relative permittivity is $\varepsilon_{r}$ = 5. The simulation parameters are shown in Table 1. In this paper, the average reward is used as the metric to evaluate system performance, defined as shown in Equation (26):

(26) $$R_{avg}=\frac{\sum_{t=1}^{T}reward(t)}{t},t=1,2,\ldots ,T$$

**Figure 5:**
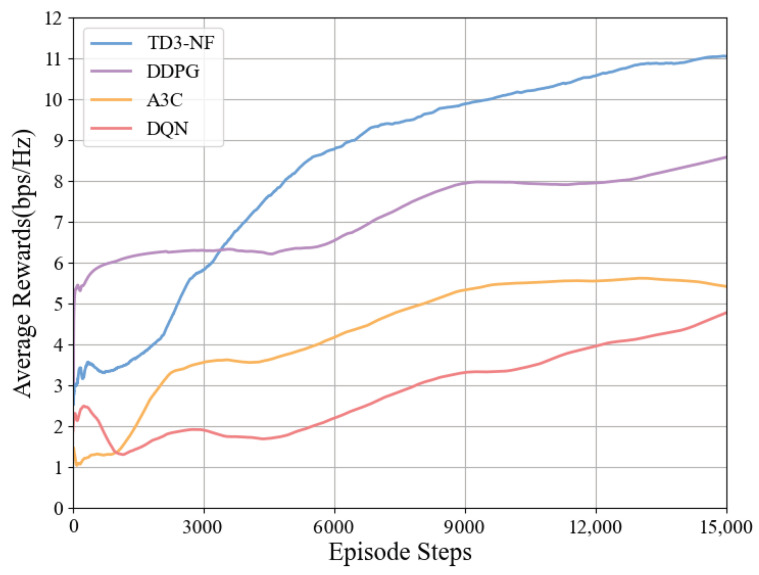
Impact of different algorithms on average reward.

As shown in Figure 5, this paper compares and analyzes the performance of four reinforcement learning algorithms (TD3-NF, DDPG, Asynchronous Advantage Actor–Critic (A3C), and Deep Q-Network(DQN)) in a RIS-assisted tunnel communication system. The experimental results indicate that the TD3-NF algorithm demonstrates the best performance, with its link rate significantly improving from the initial 2.5 bps/Hz to 11.1 bps/Hz. In contrast, the DDPG algorithm eventually stabilizes at 8.6 bps/Hz, with the performance bottleneck primarily stemming from the issue of overestimating Q-values; the learning curve of the A3C algorithm was relatively flat, increasing from approximately 1.0 bps/Hz to 5.7 bps/Hz. The DQN algorithm, constrained by the expressive capability of its discrete action space, only slowly improved from 2.3 bps/Hz to 4.8 bps/Hz. Overall, the TD3-NF algorithm emerged as the most suitable for this tunnel communication system due to its strong adaptability and efficient policy learning. In contrast, DDPG, A3C, and DQN failed to achieve comparable optimization results due to their respective limitations. These algorithms face varying degrees of performance bottlenecks, particularly when handling complex continuous or large-scale action spaces.

**Figure 6:**
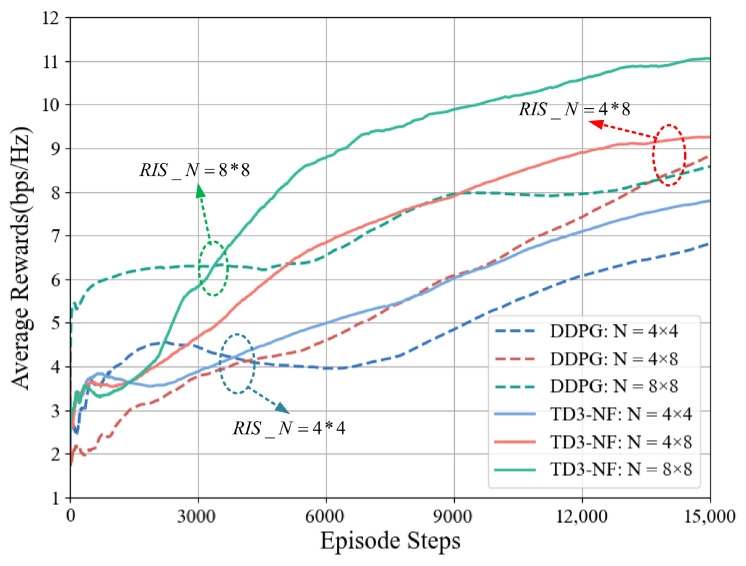
Impact of different RIS element numbers on average reward $(P_{t}$ = 38 dBm).

When $P_{t}=38$ dBm, the changes in the system average reward under different RIS arrays for the DDPG and TD3-NF algorithms are shown in Figure 6. As the number of RIS elements increases, the system average reward using the TD3-NF algorithm improves from 7.8 bps/Hz to 11.1 bps/Hz, an increase of 3.3 bps/Hz, while DDPG only improves by 1.8 bps/Hz. This indicates that the TD3-NF algorithm can more effectively boost the system’s average reward, exhibiting stronger scalability and optimization capabilities as the number of RIS elements increases.

**Figure 7:**
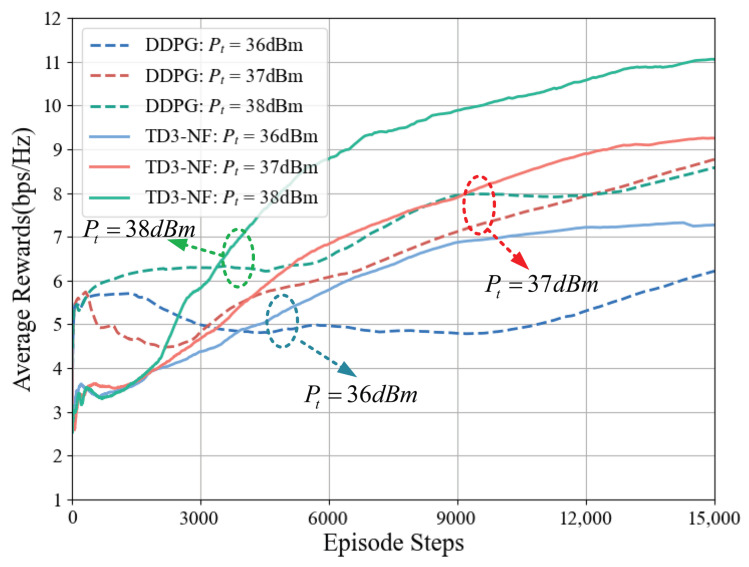
Impact of different transmission power on average reward (N=8×8).

When the number of RIS elements in the system is 64, the average reward changes for the two algorithms under different transmit power levels are shown in Figure 7. When the power increases by 2 dBm, the TD3-NF algorithm exhibits a faster reward growth rate. When $P_{t}=38$ dBm, the average reward of the system under this algorithm increases from 3 bps/Hz to 11.1 bps/Hz, an increase of 8.1 bps/Hz; while the reward growth of DDPG only improves by 3 bps/Hz.The TD3-NF algorithm consistently outperforms the DDPG algorithm in efficiency across different transmission power levels, particularly under high-power conditions, demonstrating superior adaptability and optimization capabilities in complex environments. Even under identical conditions, the TD3-NF algorithm achieves significantly higher average reward improvements compared to DDPG.

Figure 6 and Figure 7 show that the performance of the TD3-NF algorithm is more prominent when the number of RIS elements and transmission power are increased, especially under higher RIS configurations and transmission power conditions, where the learning speed and reward value are significantly improved. This indicates that optimizing the number of RIS elements and transmission power has a positive effect on algorithm performance.

**Figure 8:**
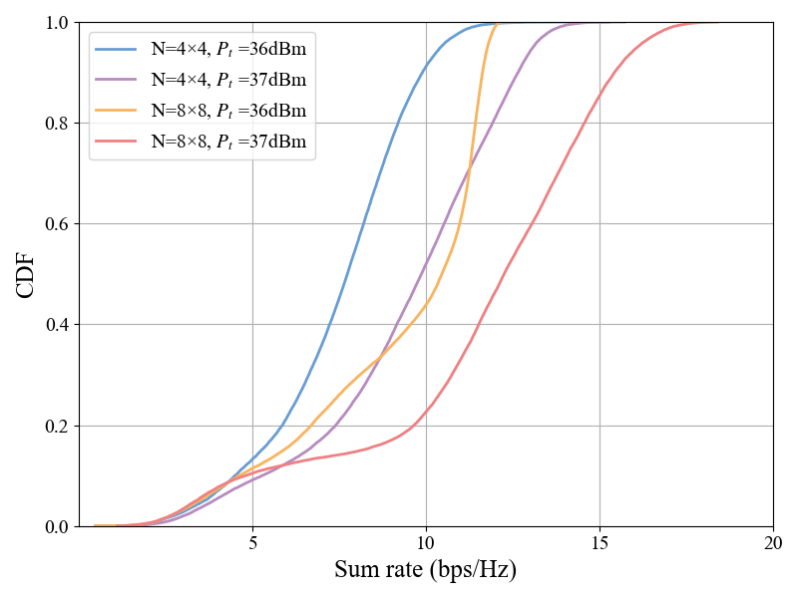
CDF of total rate for different system settings.

The CDF of the total system rate for different numbers of RIS elements and transmit powers is shown in Figure 8. As the transmit power and number of RIS elements increase, the CDF curve shifts to the right. When CDF = 0.6 and $P_{t}=37$ dBm, the number of RIS elements is positively correlated with the system rate, and the rate for an 8 × 8 array is 13.45 bps/Hz.The CDF curve validates the observations in Figure 6 and Figure 7, confirming that both the average and peak rates of the system improve with increases in transmit power and the number of RIS elements.

**Figure 9:**
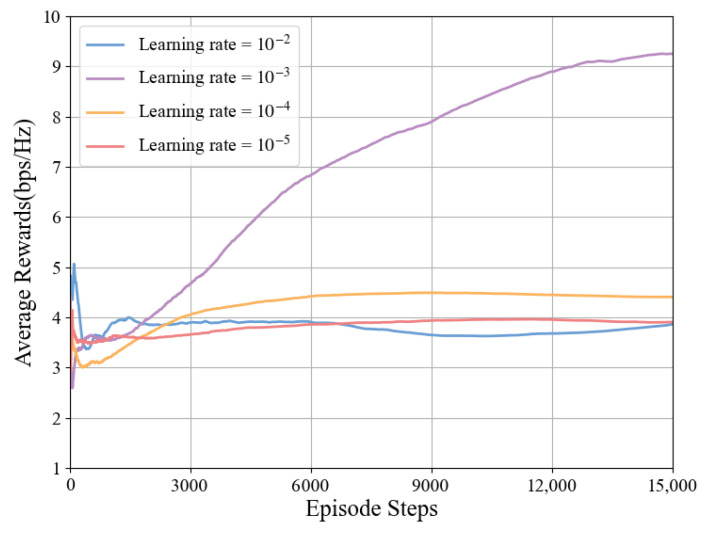
Impact of learning rate on average reward under TD3-NF algorithm.

**Figure 10:**
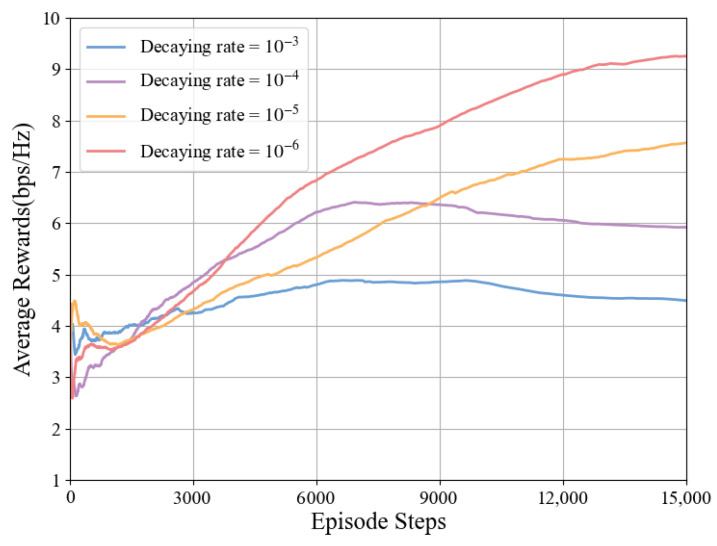
Impact of decay rate on average reward under TD3-NF algorithm.

In the algorithm proposed in this paper, the Actor and Critic networks use fixed learning rates and decay rates. Under the conditions of N=8×8 and $P_{t}=37$ dBm, the relationship between different learning rates, decay rates, average rewards, and episode steps is shown in Figure 9 and Figure 10.

The impact of different learning rates on algorithm performance is shown in Figure 9. When the learning rate is $10^{-3}$, the system achieves optimal performance, so this paper selects $10^{-3}$ as the learning rate for this model. However, when the learning rate is $10^{-2}$, the algorithm performs the worst, which may be due to the excessively high learning rate causing the algorithm to oscillate continuously during the optimization process and unable to find the optimal solution.

The relationship between the average reward and the time-step length under different decay rates is shown in Figure 10. Compared with the learning rate, the decay rate has a smaller impact on system performance. When the decay rate is $10^{-6}$, the system achieves optimal performance, while the algorithm performs worst when the decay rate is $10^{-3}$. Therefore, this paper selects $10^{-6}$ as the decay rate for this model.

## 5. Conclusions

This study proposes a communication design scheme for semi-circular arch mine tunnels based on RIS assistance. By jointly optimizing the base-station beamforming matrix and the RIS phase-shift matrix, the TD3-NF algorithm is used to evaluate the system link rate. The results show that the TD3-NF algorithm significantly outperforms traditional DDPG, A3C, and DQN algorithms in link-rate optimization, with the average link rate improving from an initial 2.5 bps/Hz to 11.1 bps/Hz. Compared to the second-best DDPG algorithm, the link rate achieved using the TD3-NF algorithm is superior to that of the DDPG algorithm under the same conditions. When the transmit power is 38 dBm and and 8 × 8 RIS elements, the system’s average link rate reached 11.1 bps/Hz. The study also analyzed the impact of RIS element count and base-station transmit power on system performance, finding that increasing the number of RIS elements and boosting transmit power can further enhance system performance, offering new solutions to improve the efficiency of intelligent mine communication systems.However, current research still faces several challenges, including performance optimization in complex mine structures, interference issues in multi-hop RIS deployment, and stability concerns with intelligent algorithms. Future research could focus on the following directions: further optimizing RIS deployment and multi-RIS collaborative optimization in complex environments, enhancing algorithm adaptability and stability in dynamic mine settings, and exploring the integrated application of RIS with other intelligent technologies. Concurrently, practical deployment and experimental validation will provide crucial evidence for further refining this solution.

## Figures and Tables

**Table 1:** Simulation parameters.

Parameter	Value
Frequency	2.4 GHz
Batch size	16
Number of episodes	$5\times 10^{3}$
Episode steps	$1.5\times 10^{4}$
Experience replay E	$10^{6}$
Discount factor γ	0.999
Learning rate	$10^{-3}$
Decaying rate τ	$10^{-6}$

## Data Availability

The original contributions presented in the study are included in the article, and further inquiries can be directed to the corresponding author.
